# Resolution of Inflammation after Skeletal Muscle Ischemia–Reperfusion Injury: A Focus on the Lipid Mediators Lipoxins, Resolvins, Protectins and Maresins

**DOI:** 10.3390/antiox11061213

**Published:** 2022-06-20

**Authors:** Cindy Barnig, Gaetan Lutzweiler, Margherita Giannini, Anne Lejay, Anne-Laure Charles, Alain Meyer, Bernard Geny

**Affiliations:** 1 Team 3072 “Mitochondria, Oxidative Stress and Muscle Protection”, Translational Medicine Federation of Strasbourg (FMTS), Faculty of Medicine, University of Strasbourg, 11 Rue Humann, 67000 Strasbourg, France; cbarnig@chu-besancon.fr (C.B.); margherita.giannini@chru-strasbourg.fr (M.G.); anne.lejay@chru-strasbourg.fr (A.L.); anne.laure.charles@unistra.fr (A.-L.C.); alain.meyer1@chru-strasbourg.fr (A.M.); 2 Physiology and Functional Exploration Service, University Hospital of Strasbourg, 1 Place de l’Hôpital, 67091 Strasbourg, France; 3 INSERM, EFS BFC, UMR1098, Interactions Hôte-Greffon-Tumeur/Ingénierie Cellulaire et Génique, University Bourgogne Franche-Comté, LabEx LipSTIC, 25000 Besançon, France; 4 Department of Chest Diseases, University Hospital of Besançon, 25030 Besançon, France; 5 Laboratoire de Polymères, Biopolymères et Surfaces (PBS), Université de Rouen Normandie, 55 Rue Saint-Germain, 27000 Evreux, France; gaetanlutzweiler@gmail.com

**Keywords:** lipid mediators, lipoxin, resolvins, protectins and maresins, inflammation, oxidative stress, ischemia/reperfusion, muscle

## Abstract

Skeletal muscle ischemia reperfusion is very frequent in humans and results not only in muscle destruction but also in multi-organ failure and death via systemic effects related to inflammation and oxidative stress. In addition to overabundance of pro-inflammatory stimuli, excessive and uncontrolled inflammation can also result from defects in resolution signaling. Importantly, the resolution of inflammation is an active process also based on specific lipid mediators including lipoxins, resolvins and maresins that orchestrate the potential return to tissue homeostasis. Thus, lipid mediators have received growing attention since they dampen deleterious effects related to ischemia–reperfusion. For instance, the treatment of skeletal muscles with resolvins prior to ischemia decreases polymorphonuclear leukocyte (PMN) infiltration. Additionally, remote alterations in lungs or kidneys are reduced when enhancing lipid mediators’ functions. Accordingly, lipoxins prevented oxidative-stress-mediated tissue injuries, macrophage polarization was modified and in mice lacking DRV2 receptors, ischemia/reperfusion resulted in excessive leukocyte accumulation. In this review, we first aimed to describe the inflammatory response during ischemia and reperfusion in skeletal muscle and then discuss recent discoveries in resolution pathways. We focused on the role of specialized pro-resolving mediators (SPMs) derived from polyunsaturated fatty acids (PUFAs) and their potential therapeutic applications.

## 1. Introduction

Acute inflammation is a protective and indispensable response of the host to tissue injury, infection or metabolic stress, that has been developed throughout evolution [1]. In physiological conditions, the acute inflammatory response is self-limited and classically divided into initiation and resolution phases with the ultimate aim of restoring tissue integrity and homeostasis.

Until the beginning of this century, acute inflammation was thought to resolve passively. Decreased expression of pro-inflammatory cytokines, dilution of chemokine gradients over time and natural catabolism of pro-inflammatory mediators likely explained the arrest of leukocyte migration from blood into tissues. However, more recent investigations focused on inflammation have shown critical molecular and cellular processes that are specifically engaged in the inflammatory response to actively promote resolution enabling tissues to restore their functions. Indeed, pro-resolving mediators have been identified that act on specific receptor targets and induce key cellular steps inducing resolution: stopping polymorphonuclear neutrophil (PMNs) recruitment, induction of leukocyte apoptosis, activation of clearance of apoptotic cells and macrophage reprogramming from a pro-inflammatory to a pro-resolving phenotype [2].

Ischemia is a pathological condition secondary to restriction or total lack of blood flow to an area or organ. Prompt reperfusion of an ischemic organ is mandatory to prevent tissue infarction and permanent loss of function. However, reperfusion may exacerbate oxidative-stress-related local tissue injury and generate remote systemic inflammation with multiple organ failure. This fast paradoxical reaction is called ischemia–reperfusion injury (IRI).

Mechanisms contributing to the pathogenesis of IRI are numerous and complex. Nevertheless, it is now clear that excessive and unresolved inflammation is one of the keystones of IRI and the identification of the dysfunctioning pro-resolution mechanisms in IRI injury is of wide interest. Besides ischemic and/or pharmacologic conditioning [3,4], more effective preventive therapy of IRI injury is still lacking and a better understanding of inflammatory mechanisms may open windows to therapeutic approaches.

If IRI can occur in almost every organ, it is critically important when it involves skeletal muscle. Although skeletal muscle is not considered a vital organ, it is vulnerable to ischemia and reperfusion of large muscular mass invariably leads to multi-organ failure and death via systemic effects [5]. IRI of skeletal muscle is a common issue in vascular surgery procedures such as extremity revascularization, vascular reconstruction and abdominal aortic aneurysm repair.

In this review, we first aimed to describe the reactive oxygen species (ROS) and inflammatory responses during ischemia and reperfusion in skeletal muscle and then discuss recent discoveries in resolution pathways. We focused on the role of (SPMs) derived from (PUFAs) and their potential therapeutic applications in skeletal muscle ischemia–reperfusion injury.

## 2. Inflammatory Response to Ischemia and Reperfusion in the Skeletal Muscle

During ischemia, the deprivation of oxygen and nutrients to the tissue triggers a series of events leading to cell injury and cell death. As oxygen levels decrease, the metabolism switches from aerobic to anaerobic with the accumulation of metabolic byproducts and cellular acidification. Metabolic demand can exceed compensatory ATP production and ATP levels fall to critically low levels. ATP-dependent membrane pumps become nonfunctional and intracellular sodium and calcium accumulate. Ionic unbalance leads to cell swelling, membrane rupture and oncotic cell death.

Prompt restoration of perfusion is crucial to limit ischemia-related damages. However, reperfusion triggers an intense local inflammation that can paradoxically exacerbate injury and cell death, especially during the first few hours of reperfusion [4,6]. Several mechanisms that temporally overlap from the beginning of the reperfusion will be responsible for the onset of IRI: mitochondrial failure and production of reactive oxygen species (ROS), release of endogenous danger molecules from necrotic and injured cells, secretion of chemokines and pro-inflammatory cytokines, activation of the complement and recruitment of neutrophils. These nonspecific signals cause vasodilation, endothelial dysfunction with increased vascular leakage followed by a self-amplifying network of pro-inflammatory pathways. Leukocytes are continuously recruited and activated [7].

Initially confined in the muscle itself, the post-ischemic inflammation is afterwards observed at remote sites as a consequence of systemic activation with potential multiple organ failure.

### 2.1. Oxidative Stress

#### 2.1.1. Deleterious Action of ROS

Aberrant production of ROS and accumulation of oxidative products within the tissues are probably the keystone initiators of IRI. Indeed, ROS are massively produced during reperfusion, as restored blood supply reintroduces a large amount of oxygen in ischemic tissues [8]. ROS have strong oxidative properties in ischemic tissues and lead to cell death via DNA damage, protein carbonylation and lipid peroxidation [9].

This crucial role of ROS in IRI is underlined by many studies that generally showed a protective effect of antioxidant therapies during ischemia–reperfusion of skeletal muscles [1,10]. Accordingly, oxidative skeletal muscles are less prone to IRI damages than glycolytic ones, thanks to their antioxidant pool [10,11,12,13,14]. 

Thus, excessive generation of ROS beyond antioxidant-scavenging capacity upon reperfusion will exacerbate the inflammatory response and lead to tissue injuries at the local level. Indeed, alteration of cellular components and oxidant formation during reperfusion can alter the microcirculation, increasing vascular permeability and formation of oedema. In addition, ROS favor a pro-inflammatory environment promoting the formation of the NLR family pyrin domain containing 3 (NLRP3) inflammasome, formation of chemotactic stimuli and pro-inflammatory cytokines (i.e., IL-1β) and expression and/or activation of adhesion molecules, which leads to neutrophil infiltration [15].

Of note, under physiological conditions, ROS also play a key role. When the ROS level is low and short-lasting, likely in relation with electron leakage from the mitochondrial electron transport chain, it contributes to normal cellular functions, including important signal transduction [15,16].

#### 2.1.2. Sources of ROS

There are several mitochondrial sources of ROS production depending on cellular conditions [17,18,19,20]. During ischemia–reperfusion, the main source of ROS may be the dysfunctioning of the mitochondrial respiratory chain. Animal models such as aortic cross-clamping or leg tourniquet induced reduction in mitochondrial oxidative capacity and increased ROS production in lower limb IR [21,22]. During IRI, the metabolic substrates accumulating at complex I will be rapidly oxidized and the accumulation of succinate will lead to a reverse electron transport from complex II to complex I, therefore increasing ROS production by complex I. Such reverse electron transport is critical, particularly in IRI [23].

The second source of ROS seems to be a dysfunctioning xanthine oxidase (XO). XO is localized in microvascular endothelial cells of skeletal muscle under an oxidized nicotinamide adenine dinucleotide (NAD)-dependent dehydrogenase form in nonischemic cells. During ischemia, xanthine dehydrogenase is converted to xanthine oxidase in hypoxic endothelial cells. When oxygen is supplied in large amounts during reperfusion, electrons from xanthine oxidase are transferred to molecular oxygen, forming large quantities of superoxide anions [24].

In addition to XO and mitochondrial respiratory chain in endothelial and muscle cells, the activation of neutrophils will generate ROS by nicotinamide adenine-dinucleotide phosphate (NADPH) oxidase [25].

### 2.2. Mitochondrial Failure

The role of mitochondria extends far beyond ROS generation. The mitochondrial permeability transition pore (mPTP) is activated under ROS exposure and increases mitochondrial calcium levels [26], leading to the disruption of the electrochemical gradient, uncoupling of oxidative phosphorylation and ATP depletion. In addition, the increase in osmotic pressure leads to mitochondrial swelling and membrane rupture with necrosis or apoptosis [26].

### 2.3. Endogenous Danger Molecules

IRI shares many phenotypic parallels with the immune response toward invading microorganisms.

Indeed, several danger-associated molecular patterns (DAMPs) are released by injured and necrotic cells during reperfusion including high-mobility group box-1 (HMGB1), heparan sulfate, adenosine triphosphate (ATP), nuclear DNA and RNA [27,28]. The mitochondria will also release DAMPs including mitochondrial DNA (mtDNA) and N-formyl peptides [28].

DAMPS are recognized by highly conserved host receptors, called pattern recognition receptors (PRRs), that also mediate the immune response to microorganisms. This heterogeneous group of receptors includes Toll-like receptors (TLR), nucleotide oligomerization domain-like (NOD) receptors (NLR), C-type lectin receptors (CLR) and RIG-I-like receptors (RLR). PRRs are expressed by all cells of the innate immune system (neutrophils, monocytes, macrophages, dendritic cells and natural killer) [29] and some non-immune cells such as endothelial and epithelial cells.

Ligation of PRRs leads to the activation of downstream signaling pathways (nuclear factor kappa B (NF-κ B), mitogen-activated protein kinase (MAPK) and type I interferon) with the induction of pro-inflammatory cytokine and chemokine secretion [30].

Interestingly, oxidative stress may upregulate TLR4 expression and prime inflammatory cells for increased responsiveness to subsequent stimuli [31].

In particular, formylated peptides and mDNA released from mitochondria are structurally similar to bacterial components. In skeletal muscle necrosis, these mitochondrial DAMPs promote neutrophil activation and their recruitment to inflamed tissues [32].

### 2.4. Chemokines and Cytokines

Ischemia reperfusion-induced inflammation is characterized by the production of cytokines and chemokines. Leukocytes and endothelial cells seem to be the major sources of cytokines production [33]. Cell injury activates cyclooxygenase and lipoxygenase pathways along with transition metal ions, which increases lipid peroxidation in the surrounding tissues. Products of the lipoxygenase pathway are responsible for neutrophil activation.

After the activation of the PRRs by DAMPS in response to ischemia–reperfusion of skeletal muscles, the production of interleukin 1β (IL-1 β), IL-6, tumor necrosis factor α (TNF-α), monocyte chemoattractant protein 1 (MCP-1) and IL-8 is induced [25]. The roles of interleukin-1 (IL-1), interleukin-6 (IL-6) and tumor necrosis factor α (TNF-α) are well documented in IRI injury [10,34]. These cytokines provide signals between the responding leucocytes and the vascular endothelial barrier and are believed to be responsible for the accumulation and activation of leukocytes [35,36,37].

### 2.5. Neutrophils and Pro-Inflammatory Macrophages

Neutrophils are the largest circulating fraction of leukocytes. After reperfusion, neutrophils are the first leukocytes to arrive at the site of injury.

During injury, neutrophils are mainly recruited by chemokines produced by tissue-resident immune cells and endothelial cells [38]. Most evidence supports the view that mononucleated cells that normally reside in muscles are activated by the injury, and then secrete chemotactic signals to circulating inflammatory cells [39].

The role for neutrophils in the promotion of muscle damage soon after muscle injury is now clearly established [25,40,41,42,43,44]. In experimental IRI models, the inhibition or depletion of neutrophils before ischemia–reperfusion protects from muscle damage [45,46,47,48,49,50,51,52,53]. These data suggest that neutrophil accumulation into postischemic tissue is a cause rather than an effect of reperfusion injury.

Rolling and tight adhesion of neutrophils are initiated through the upregulation of adhesion molecules on endothelial cells. Once they transmigrate through the endothelium, neutrophils are able to damage the injured muscle and healthy bystander tissues by the release of proteases and ROS (‘respiratory burst’) [53]. Cytotoxicity assays in which muscle cells were cocultured with activated neutrophils showed that neutrophil lysis of muscle cells could be largely prevented by the addition of superoxide dismutase (SOD) [54].

Moreover, neutrophils release interleukins and leukotrienes that attract and activate additional leukocytes and produce neutrophil extracellular traps (NETs) by releasing nuclear chromatin and granule proteins. Their implications have been demonstrated in TLR4 knock-out (KO) mice with decreased NET levels showing reduced muscle damage subsequent to hind limb injury [55]. Neutrophil products also have a role in complement activation [56].

The early wave of polymorphonuclear neutrophils (PMNs) is followed by the recruitment of blood monocytes, which differentiate into macrophages, sources of pro-inflammatory cytokines. Macrophages can promote muscle damage in vivo and in vitro through the release of free radicals [57]. Cytotoxicity assays have shown that macrophages lyse target muscle cells by a nitric oxide (NO)-dependent, superoxide-independent mechanism and that their cytolytic capacity is increased by the presence of neutrophils [54]. However, the prevalence of this pro-inflammatory phenotype is gradually superseded by the emergence of a more anti-inflammatory, proregenerative phenotype [57].

### 2.6. Activation of Complement

Complement system is one of the mediators of innate immune systems. Three pathways can initiate the complement system: classical, alternative and mannose binding lectin (MBL) pathways. They are composed of circulating precursor proteins that can be enzymatically activated. While the activation of each pathway is distinct, they converge to the activation of C3 convertase, leading to the formation of the membrane attack complex (MAC) that is capable of forming a lytic pore into the target’s ischemic cell wall [58].

Complement increases vascular permeability, oedema generation and infiltration of leukocytes. Moreover, several animal models demonstrated a key role of complement activation in inducing lesions in remote organs (e.g., lung and liver) after IRI of skeletal muscle [51,59,60].

All three pathways are thought to be activated in IRI of skeletal muscle, but the classical and the MBL pathways seem to have a critical role. Indeed, in humans, the role of complement activation has been demonstrated by increased C3a and C5a serum concentrations after lower extremity ischemia [61,62]. During reperfusion, lysis of parenchymal cells by the MAC [63], recruitment of leukocytes through chemotaxis by C3a and C5a and production of inflammatory cytokines and chemokines [64] are also implicated in IRI.

## 3. Principles of Resolution of Inflammation

A new paradigm considers the resolution of inflammation as a coordinated and active process aiming to restore tissue integrity and function [65]. If inflammation is not stopped and/or overwhelming, it may have debilitating consequences as encountered in IRI.

Several cellular mechanisms favor the repair of inflamed or injured tissues and restore homeostasis. First, PMN recruitment will cease and chemokine and cytokine gradients are counter-regulated. After having performed their action at the inflamed site, neutrophils undergo apoptosis. Then, monocytes are recruited non-phlogistically and death signals induce cell death or apoptosis of the inflammatory granulocytes, partly mediated through natural killer (NK) cells [66]. Apoptosis of neutrophils, in contrast to necrosis, will prevent bystander tissue injury occurring from the release of the potentially toxic cellular constituents. In the third phase, phagocytosis of apoptotic neutrophils by macrophages will prompt a switch from pro- (M1) to anti-inflammatory (M2) macrophage phenotype. This initiates the repair of tissue architecture and function in response to anti-inflammatory cytokines, growth factors and pro-resolving mediator release. When phagocytosis is realized, draining blood or lymph vessels (egress) allow macrophages to leave the inflammation site.

A large, growing class of molecules with pro-resolving functions promotes many of those cellular processes. These mediators are produced during the inflammatory response in a coordinated temporal and spatial manner, in order to restrain and resolve inflammation. Interestingly, unlike anti-inflammatory mediators, pro-resolving mediators are not immunosuppressive but rather tissue protective and reparative [67].

Enhancing the host immune response, they promote key cellular steps of resolution of inflammation. Thus, these molecules favor a non-phlogistic recruitment of monocytes (without pro-inflammatory mediators), granulocyte apoptosis, macrophage phagocytosis of apoptotic cells, switch of macrophages and the return of non-apoptotic cells to lymphatics and blood vessels. They also stimulate tissue regeneration, allowing the restitution of barrier integrity [68,69].

Pro-resolving mediators are of a different nature and many display overlapping functions. They include SPMs (lipoxins, resolvins, protectins and maresins), proteins and peptides (annexin A1 (AnxA1), galectins, adrenocorticotropic hormones (ACTH) and IL-10), gaseous mediators including hydrogen sulfide (H2S) and carbon monoxide (CO), nucleotides (e.g., adenosine) and neuromodulators released under the control of the vagus nerve such as acetylcholine and neuropeptides released from non-adrenergic non-cholinergic neurons [70]. There is now increasing evidence that ROS, beyond the initiation and the progression of the inflammatory response, are also involved in the resolution at later steps of the inflammatory response [71].

The mediators of resolution can be produced and act locally in paracrine and autocrine manners and they can be produced at remote sites, acting through their systemic release and extravasation to sites of inflammation.

Some evidence from animal models suggests that different cellular and biological mechanisms involved in resolution (i.e. the clearance of apoptotic cells by macrophages, production of pro-resolving mediators) are impaired with age [72].

## 4. Specialized Pro-Resolving Lipid Mediators

Experimental models of acute inflammation that are self-limiting and naturally resolved have led to the identification of a novel family of SPM generated from PUFAs.

SPMs are generated via lipoxygenase (LOX)-catalyzed reactions. Most of the SPMs are produced during intercellular interactions between various leukocytes (neutrophils, macrophages, etc.) and other cells such as epithelial and endothelial cells, expressing the required biosynthetic and compartmentalized enzymes and sharing intermediate metabolites bidirectionally [73]. Among leukocytes, neutrophils are key producers of SPMs, including lipoxins, leading members of the SPM family [74]. While structurally and functionally distinct from prostaglandins and leukotrienes, lipoxins are also products of omega-6 arachidonic acid (AA) (20:4n − 6) present as phospholipids in cell membranes. When the cells are activated, specific phospholipase A2 (PLA2) enzymes liberate AA from the sn-2 fatty acyl bond of these phospholipids [75].

Moreover, during acute inflammation, SPM biosynthesis in leukocytes is initiated by lipid-mediator class switching. Early pro-inflammatory lipid mediators, such as prostaglandins and leukotrienes, are pivotal and activate the biosynthetic machinery for the later conversion of PUFA to SPMs [74]. For example, human PMNs exposed to PGE2 or PGD2 induce 15-lipoxygenase (15-LOX) switch their phenotype from LTB_4_ production to lipoxin production [74]. Macrophages also have the capacity to produce SPMs, including lipoxins [73]. In addition to AA-derived lipoxins, the omega-3 PUFAs eicosapentaenoic acid (EPA, C20:5n − 3) and docosahexaenoic acid (DHA, C22:6n − 3) can be enzymatically converted by LOX-catalyzed reactions to SPMs, including resolvins, protectins and maresins. Although EPA and DHA are merely synthesized in humans, they are components of seafood, particularly oily fish, liver oil, krill oil and algal oil supplements and of a small number of highly concentrated pseudopharmaceutical products [70]. SPMs shows biological effects in the nanomolar range and signal through cognate receptors expressed by a wide range of cell types, including immune and structural cells. To date, four human SPM receptors are identified: ALX/FPR2, ERV1, resolvin D1 receptor (DRV1) and resolvin D2 receptor (DRV2). While identified depending on the ligand used (LXA4, RvE1, RvD1 and RvD2, respectively), each receptor is capable of interactions with additional SPMs [76].

Globally, the pro-resolving actions of SPMs include the stimulation of non-phlogistic (without elaborating pro-inflammatory mediators) recruitment of monocytes, inducing granulocyte apoptosis, enhancing macrophage phagocytosis of apoptotic cells, switching macrophages from classically to alternatively activated cells, promoting the return of non-apoptotic cells to the lymphatics and blood vessels and stimulating tissue regeneration to restitute barrier integrity [2]. During efferocytosis, SPM production is further increased, enhancing the clearance of debris and apoptotic cells through autocrine and paracrine pathways.

## 5. Resolution Mechanisms in IRI: Involvement of Immune Cells and of Specialized Pro-Resolving Mediators

**Figure 1:**
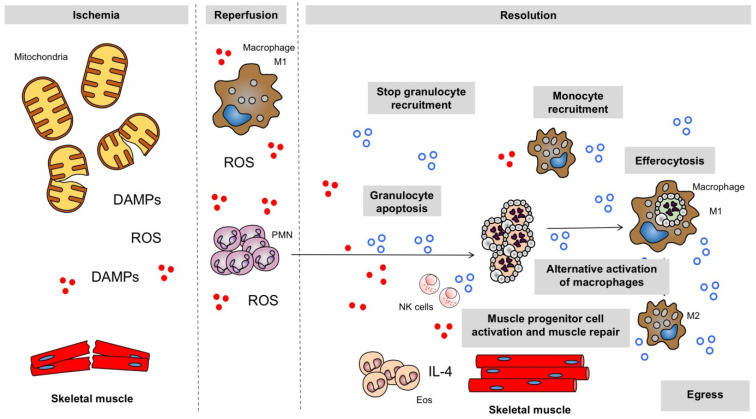
Resolution of inflammation in ischemia–perfusion injury in skeletal muscle.

Mechanisms of inflammation resolution are of major importance in IRI (Figure 1). During the early phase of reperfusion after ischemia, the accumulation of neutrophils has to be tightly controlled as too many granulocytes can promote uncontrolled inflammation with local and remote tissue injury. Moreover, rapid clearance of inflammatory leukocytes is essential for a successful resolution response and the restoration of function within the skeletal muscle following IRI.

During the initial state of perfusion after ischemia, a vast array of pro-inflammatory mediators (closed red circles) are secreted (ROS) and danger-associated molecular patterns (DAMPs) are released. The resolution of inflammation is now recognized as an active process brought about by the biosynthesis of active endogenous anti-inflammatory and pro-resolving mediators released from various cell types (open blue circles), which act on key cellular events of inflammation to promote the return to homeostasis. PMN: neutrophil; NK cells: natural killer cells; IL-4: interleukine-4; Eos: eosinophils; M1: classically activated macrophages; M2: alternatively activated macrophages.

### 5.1. Macrophages

Following skeletal tissue injury, circulating classically activated (M1) monocytes infiltrate the involved muscle and promote inflammation through cytokine production and ROS release. Depletion of macrophages (MP) prior to IRI is known to reduce injury in an experimental murine kidney IRI model [77].

However, macrophages with pro-resolving functions present at later stages of the inflammatory response are important to direct the outcome. Indeed, when macrophages are depleted or their migration is blocked through genetic or pharmacological means days after the onset of ischemia, a delayed resolution of skeletal muscle inflammation is observed [78]. Moreover, severe reduction in muscle recovery from ischemia occurs with the genetic deletion of CCR2 and monocyte chemoattractant protein-1 (MCP-1), which both substantially impair MP recruitment [79]. Phagocytosis of tissue debris is a crucial function of MPs in the resolution of inflammation [80,81,82]. Mouse strains characterized by slower rates of phagocytic removal of muscle debris appear to have slower rates of muscle regeneration [83]. Moreover, phagocytose of apoptotic neutrophils and myofiber debris by macrophages triggers a switch to an alternatively activated anti-inflammatory (M2) phenotype [39,79]. M2 macrophages are able to modulate muscle progenitor cell activation and coordinate muscle repair by producing a large variety of pro-resolving mediators, including SPMs [73]. During the later stages after IRI of the limb, these M2 populations are predominant [84]. If distinct macrophage populations are related to separate phases of muscle recovery [85,86], hypoxia-inducible factors (HIFs) in macrophages do not seem to be implicated in skeletal muscle regeneration [87].

In conclusion, the current data indicate that pro-inflammatory M1 macrophages are involved in early tissue damage during ischemia and M2 macrophages will have pro-resolving functions later on during the inflammatory response after reperfusion. A tight balance between M1 and M2 macrophages is therefore needed.

### 5.2. Eosinophils

Eosinophils are another key factor of the innate immune cell population, active within the resolution phase of inflammation. They were recently proven to be indispensable in the muscle regenerative response to injury. Eosinophils are target cells of a wide range of pro-resolving mediators but are also able to produce pro-resolving mediators during the inflammatory response [70]. For example, eosinophils are rapidly recruited during skeletal muscle injury and they are required for the proliferation of muscle-resident fibro/adipocyte progenitors through the secretion of IL-4 [88]. The binding to high-mobility group box-1 (HMGB1), a necrosis signaling molecule, by the receptor for advanced glycation end products (RAGE) expressed on eosinophils can mediate the chemotactic migration of eosinophils and their response to tissue injury or necrosis [89]. Healing is favored by eosinophils through actions on the vasculature and elevating epithelial cell proliferation [90], releasing vascular endothelial growth factor (VEGF), fibroblast growth factor (FGF) and transforming growth factor-β1 (TGF-β1) [91] as well as osteopontin [90].

### 5.3. Specialized Pro-Resolving Mediators (SPMs)

Because SPMs control unwarranted PMN accumulation and promote their removal, SPMs may be of interest for tissue recovery during ischemic injury. First, lipoxins prevent oxidative-stress-mediated tissue injury [92,93,94,95]. Moreover, SPM precursors, including EPA-derived 18-HEPE and 15-HEPE, are produced by vascular endothelial cells during hypoxia [96]. Under hypoxia, human PMN RvE2 biosynthesis is also enhanced [97] and IR in the kidneys results in the biosynthesis of D-series resolvins and protectins [98].

Recent studies on experimental models show the protective roles of SPMs in tissue injury in IRI disease models more specifically.

Many of these animal models show that treatment with SPMs prior to ischemia decreases PMN infiltration [98,99,100]. The endogenous protective role of SPMs was also observed in mice without the *Alx/Fpr2 or the Gpr18* (also known as DRV2) receptors, in which IR resulted in excessive leukocyte accumulation [100,101]. During injury, SPM receptor signaling induced by one mediator can also upregulate the expression of additional SPMs, further activating other SPM receptors. Thus, RvD2-DRV2 induced RvD5 and PD1 for resolution [102].

As a perspective, one approach to overcome tissue loss after IRI relies on the implantation of biomaterials in order to restore or regenerate the functions of damaged muscles. In particular, porous scaffolds can provide space for cell colonization, proliferation and neo-vessel formation, followed by its gradual dissolution. Hydrogels are of prime interest as they provide a well-hydrated environment along with an extracellular matrix-like architecture, suitable to sustain the functions of resident cells (98). Biomaterials can also participate in the modulation of the inflammatory process mostly through carrying bioactive molecules including growth factors and cytokines which are subsequently exposed to the adherent cells or passively released from the material. For instance, alginate hydrogels loaded with growth factors including VEGF or insulin growth factor-1 (IGF1) allowed for a better recovery of the functions of hind-limb muscles in rodents following IRI [103,104,105]. Moreover, core–shell microparticles could be designed to achieve a stimuli-responsive oxygen delivery to mesenchymal stem cells (MSCs), which further stimulates proliferation, vascularization and paracrine secretions to induce the regeneration of skeletal muscles [106]. The use of SPMs in the biomaterial context is still limited and unexplored in the specific case of IRI since their stability in classical cell culture media can be impaired [107], along with the hydrophobicity of such molecules, which restricts their loading onto material surfaces. This issue can be lifted by core–shell particles as described by Wang et al. [108] who encapsulated lipoxin A4 into poly(lactic-co-glycolic) acid (PLGA) microspheres to achieve a gradual release. However, the processing conditions led to substantial loss of lipoxin. Some examples recently highlighted the ability of SPMs to drive the phenotypic switch from classically to alternatively activated macrophages, allowing better integration of biomaterials and triggering the restoration of tissues. This was shown for mice subjected to the implantation of a poly(ethylene) glycol (PEG)-based hydrogel loaded with IL-10 and pro-resolving lipid mediator aspirin-triggered resolvin-D1 (AT-RvD1) [109]. Poly(caprolactone) (PCL)-based vascular grafts functionalized with AT-RvD1 allowed the recruitment of CD49d+ neutrophils at the implant site which are actively involved in vascular remodeling and neovessel formation [110]. The regenerative potential of resolvin D1 embedded into a chitosan scaffold was also demonstrated in a femoral defect in rats after 2 months, the extent of bone formation was found to be higher compared to animals implanted with chitosan scaffolds solely [111].

Although there is plenty of evidence supporting the potent role of SPMs in the resolution of inflammation, the signature profile of SPMs is not straightforward to detect since these mediators such as maresin-1 or lipoxin-A4 are secreted in tiny amounts (i.e., in the nanomolar to the picomolar range) [112]. Accordingly, the techniques employed to detect and quantify SPMs need to be carefully chosen; for instance, as these small concentrations are close to the resolution limits of mass spectrometers, the ELISA method proved to not be very selective between different isomers [113]. Furthermore, the exact mechanism and the receptors involved in the SPM signaling pathways still need to be ascertained in knock-out mice [114].

## 6. Conclusions

IRI in the skeletal muscle is a serious condition with potentially local and general devastating consequences that remain a common concern in vascular surgery. During reperfusion, blood flow is again supplied to the ischemic muscle but regulatory mechanisms appear to not be totally effective to avoid cell deaths [115]. IRI manifests as a paradoxical unadapted inflammation with the release of ROS, breakdown of lipidic cell membranes and attraction of neutrophiles and activation of the complement system. Currently, a very effective preventive therapy of IRI is still lacking. Most commonly used strategies try to limit pro-inflammatory pathways by means of pharmacological intervention, hypothermia, hyperbaric oxygen or pre/post-injury conditioning. Those conventional anti-inflammatory strategies have two main drawbacks: they are only partially effective they and nonspecifically blunt the production of inflammation “initiators” with potential immunosuppression.

It appears that, in IRI, the problem is not mainly how inflammation starts, but why it fails to resolve. Therefore, another promising approach to prevent IRI may be an early promotion of pro-resolving pathways. Pro-resolving mediators resolve inflammation without compromising host defense and stimulate distinct processes necessary for tissue repair and regeneration.

To date, only limited data for pro-resolving mechanisms during IRI exist. Interestingly however, in severe sepsis, a similar condition with uncontrolled systemic inflammation, producing organ dysfunction, pro-resolving pathways are activated [116]. In a randomized trial, immunonutrition increased RvE1 in patients undergoing hepatobiliary surgery, giving lower rates of infection complications and severity [117]. Accordingly, increased dietary intake of EPA and DHA—substrates for the biosynthesis of potent SPMs such as resolvins, protectins, and maresin—results in their enrichment in the blood and in many cells and tissues [118].

Although studies already reported a role of SPMs in skeletal muscle IRI (Table 1), [95,98,99,100,119,120,121] and on other IRI models (Table 2), [98,122,123,124,125] further work are warranted to determine whether enhancing SPMs via substrate supplementation or locally targeted therapy associated with biomaterials and SPM release might potentially prevent or at least mitigate the exacerbated ROS and inflammatory responses following IRI and in turn restore the functions of damaged skeletal muscles.

## Figures and Tables

**Table 1:** Role of specialized pro-resolving mediators in skeletal muscle IRI disease models.

Mediator	Disease Model	Action(s)	Ref
Lipoxin A_2_/ATL	Mouse/hind-limb IRI	Attenuate hind-limb IRI—induced lung injuryDetachment of adherent PMN in mesenteric IRI	[119]
Lipoxin A_2_	Rat/hind-limb IRI	Decreases inflammatory response, oxidative stress and cell apoptosis.	[95]
Resolvin D1	Mouse/ hind-limb IRI	Enhances perfusion recovery during ischemia,	[120]
Resolvin D1	Mouse/hind-limb IRI	Reduces PMN recruitment into the lungs	[99]
Resolvin D2	Mouse/hind-limb IRI	Reduces PMN recruitment into the lungs	[100]
Resolvin D2	Mouse/CTX-induced muscle injury	Enhances macrophage M2 efferocytosis	[121]
Protectin D1	Mouse/kidney IRI	Protects from ischemia–reperfusion-induced kidney damage and loss of function; Regulates macrophage M1 function	[98]

**Table 2:** Role of specialized pro-resolving mediators in other IRI disease models.

Mediator	Disease Model	Action(s)	Ref
Lipoxin A_2_	Rat/superfusing of the mesentery	Inhibits PMN rolling and adherence in mesenteric circulation	[122]
Lipoxin A_2_	Rat/Intestinal IRI	Attenuates intestinal ischemia–reperfusion injury	[123]
Resolvin E1	Rat/Cardiac IRI	Cardioprotective; Limits infarct size	[124]
Resolvin D1	Mouse/kidney IRI	Protects from ischemia–reperfusion-induced kidney damage and loss of function; Regulates macrophage M1 function	[98]
Resolvin D1	Rat/Cardiac IRI	Reduces accumulation of PMN and fibrosis; Leads to improved cardiac function	[125]
Protectin D1	Mouse/kidney IRI	Protects from ischemia–reperfusion-induced kidney damage and loss of function; Regulates macrophages	[98]
Resolvin D1	Mouse/kidney IRI	Decreases PMN infiltration and tissue fibrosis; Organ protective	[98]
Protectin D1	Mouse/kidney IRI	Protects from ischemia–reperfusion-induced kidney damage and loss of function; Regulates macrophage M1 function	[98]
